# COVID‐19 vaccination did not improve employee mental health: A prospective study in an early phase of vaccination in Japan

**DOI:** 10.1002/npr2.12250

**Published:** 2022-04-12

**Authors:** Natsu Sasaki, Reiko Kuroda, Kanami Tsuno, Kotaro Imamura, Norito Kawakami

**Affiliations:** ^1^ 13143 Department of Mental Health Graduate School of Medicine The University of Tokyo Tokyo Japan; ^2^ 13143 Division for Environment, Health, and Safety The University of Tokyo Tokyo Japan; ^3^ 38284 School of Health Innovation Kanagawa University of Human Services Yokosuka Japan

**Keywords:** anxiety, depression, epidemiology, occupational health, prevention, public health, SARS‐CoV‐2, vaccine

## Abstract

**Objectives:**

This study aimed to examine the effectiveness of vaccination to improve mental health among employees in Japan based on a prospective study.

**Methods:**

The data were retrieved from the Employee Cohort Study conducted during the COVID‐19 pandemic in Japan (E‐ COCO‐J) at T1 (4–10 February 2021) and T2 (22–29 June 2021). Psychological distress was measured by using an 18‐item scale of the Brief Job Stress Questionnaire (BJSQ). The analytic sample was limited to individuals employed at both T1 and T2 without missing covariates. Vaccination status was measured at T2. Statistical analysis was conducted to test the differential change in the psychological distress at T1 and T2 with the time × group interactions by using repeated ANOVA, adjusting for the covariates (gender, age, marital status, education, chronic disease, company size, industry, and occupation).

**Results:**

Of the total sample (N = 948), 105 (11.1%) were vaccinated at least once at T2. The crude mean scores of psychological distress at T1 and T2 were 41.8 and 42.0 for vaccinated participants and 41.2 and 41.2 for nonvaccinated participants, respectively, with no significant effect of having been vaccinated (Cohen's d = 0.02, *P* = 0.833). After adjusting the covariates, there was no significance (*P* = 0.446).

**Conclusions:**

The COVID‐19 vaccination was supposed to have a limited effect on mental health among Japanese employees in an early phase of vaccination. To keep providing mental health care for employees is important even after starting the vaccination program.

Deteriorated mental health is a major public health concern during the COVID‐19 outbreaks among working populations, including healthcare workers (HCWs) and non‐HCWs, and the community overall.^1^ Vaccination is considered an effective individual‐based preventive measure to control the COVID‐19 outbreaks.^2^ People adopted individual‐based preventive measures, such as hand‐washing, wearing masks, and others, to cope with the COVID‐19‐related stress.^3^ A previous study showed using a COVID‐19 contact tracking app as an individual‐based measure was associated with better mental health.^4^ The effect of the COVID‐19 vaccination as an individual‐based preventive measure on mental health is still controversial: some studies suggested the associations of vaccination on better mental health in public residence,^5^, ^6^, ^7^ but another cross‐sectional study in HCWs showed vaccinated nurses significantly presented higher anxiety than those nonvaccinated,^8^ and one Japanese study in HCWs suggested that those with high distress at baseline were likely to receive vaccines and better changes in mental health can be observed as a result.^9^ The effect of the COVID‐19 vaccination on mental health among employees, who may have more distress than community‐dwelling people, needs to be examined. We thus report a preliminary finding on whether vaccination effectively improves mental health among employees in Japan based on a prospective study.

The data were retrieved from the Employee Cohort Study conducted during the COVID‐19 pandemic in Japan (E‐ COCO‐J).^10^, ^11^ The Research Ethics Committee of The University of Tokyo approved this study (No. 10856‐[2][3][4][5]). We measured psychological distress using an 18‐item scale of the Brief Job Stress Questionnaire (BJSQ) [possible range: 18–72]^12^ at T1 (4–10 February 2021) and T2 (22–29 June 2021). Psychological distress was composed of lack of vigor, anger‐irritability, fatigue, anxiety, and depression. Screening ability to detect severe mental illness in the Japanese population has been testified.^13^ We limited the analytic sample to individuals employed at both T1 and T2 without missing covariates. Statistical analysis was conducted to test the differential change in the psychological distress at T1 and T2 with the time x group interactions by using repeated ANOVA, adjusting for the covariates (gender, age, marital status, education, chronic disease, company size, industry, and occupation). The covariates were selected because of their potential associations both with the vaccination experience and with the mental health.^14^, ^15^ In Japan, priority vaccination for HCWs began on February 17, followed by people over 65 and people with chronic diseases. Vaccination programs started on June 21 in a few workplaces.

Of the total sample (N = 948), 105 (11.1%) were vaccinated at least once at T2. Participants vaccinated at least once were more likely to be females, employed in a company with 300–999 employees, work in the medical and welfare industry, and attain college/vocational education. Proportions of vaccinated participants were healthcare workers (HCWs; n = 56/103:54.4%), non‐HCWs (n = 47/817:5.8%) and participants with unknown occupation (n = 2/28:7.1%).

The crude mean scores (standard deviations, SDs) of psychological distress measured by the BJSQ [possible range: 18–72] at T1 and T2 were 41.8 (10.9) and 42.0 (11.9) for vaccinated participants and 41.2 (11.4) and 41.2 (11.6) for nonvaccinated participants, respectively, with no significant effect of having been vaccinated (Cohen's d = 0.02, *P* = 0.833 for the time x group interaction by repeated ANOVA). After adjusting for gender, age, marital status, education, chronic disease, company size, industry, and occupation (HCWs or non‐HCWs), the estimated mean scores (standard errors, SEs) of psychological distress at T1 and T2 were 41.4 (1.7) and 42.4 (1.8) for vaccinated participants and 43.3 (1.2) and 43.4 (1.2) for nonvaccinated participants, respectively, with no significant effect (*P* = 0.446). There was no significance in any of the subscales of the BJSQ, lack of vigor, anger‐irritability, fatigue, anxiety, or depression.

Our study did not show improved psychological distress among participants vaccinated at least once compared with nonvaccinated individuals immediately after vaccination started in Japan. The findings suggest that vaccination for COVID‐19 did not improve workers' mental health in the early phase of the vaccination program. Since the worsened and sustained psychological distress during COVID‐19 outbreaks was reported,^10^ workplace measures for promotion of mental health and prevention of mental health problems among workers should be continued. An effort to continuously monitor and improve workers' mental health during the COVID‐19 should continue despite vaccination.

A previous study reported that people who had higher stress are more likely to use individual‐based preventive behaviors (eg, washing hands).^16^ There can be associations between psychological distress and intention to be vaccinated. However, since we adjusted for psychological distress before the vaccination in our study, this expectation does not apply to our findings. Another possible reason is that the study was conducted in an early phase of vaccination in this country. People may not be fully convinced that the vaccination effectively reduces the risk of COVID‐19 infection. In addition, employees' mental health may be affected not only by a perceived risk of COVID‐19 infection but also by social and economic problems due to the outbreaks.

Several limitations should be addressed. The sample was small, comprising full‐time employees recruited via an online platform in Japan. HCWs included employees working in nonclinical settings. Only a few participants were vaccinated, and some were vaccinated only once. Analyzing samples of fully vaccinated individuals in other countries and another phase of outbreaks may lead to different conclusions. Participants with missing information on covariates or who did not respond to T1 or T2 were not included in the analysis, leading the sampling bias and limited generalizability.

In conclusion, COVID‐19 vaccination was supposed to have a limited effect on mental health among Japanese employees. Therefore, to keep providing mental health care for employees is important even after starting the vaccination program.

## CONFLICT OF INTEREST

NK reports grants from SB AtWork Corp, Fujitsu Ltd, and TAK Ltd, personal fees from the Occupational Health Foundation, SB AtWork Corp, RIKEN, Japan Aerospace Exploration Agency (JAXA), Japan Dental Association, Sekisui Chemicals, Junpukai Health Care Center, Osaka Chamber of Commerce and Industry, outside the submitted work.

## DISCLOSURE

Approval of the research protocol: This study was approved by the Research Ethics Committee of the Graduate School of Medicine/Faculty of Medicine, The University of Tokyo, No. 10856‐(2)(3)(4)(5).

## AUTHOR CONTRIBUTIONS

NK was in charge of this study, supervising the process and providing his expert opinion. NS and NK organized the study design and analyzed the data. Collaborators RK, KT, and KI ensured that questions related to the accuracy or integrity of any part of the work were appropriately investigated and resolved. All authors participated in conducting the survey. NS and NK wrote the first draft of the manuscript, and all other authors critically revised it. All authors approved the final version of the manuscript.

## INFORMED CONSENT

Online informed consent was obtained from all participants with full disclosure and explanation of the purpose and procedures of this study. We explained that their participation was voluntary, and they can withdraw consent for any reason, simply by not completing the questionnaire.

## Data Availability

The data that support the findings of this study are available in a public repository (https://figshare.com/articles/dataset/E‐COCO‐J_data_for_vaccinated_status_and_mental_health/19316159).
